# Mechanisms Underlying the Impact of Feed-to-Gain Ratio Differences on Nutrient Metabolism in Simmental and Simmental × Hereford Crossbred Cattle Fed a Low-Energy Diet

**DOI:** 10.3390/ani16132068

**Published:** 2026-07-04

**Authors:** Yi Wu, Hao Chen, Danling Zhang, Lina Wang, Qi Liu, Chunjie Wang, Aorigele Chen, Hairong Wang

**Affiliations:** 1 College of Animal Science, Inner Mongolia Agricultural University, Hohhot 010018, China; m18247812243@163.com (Y.W.); chenhao9781@126.com (H.C.); 15326036216@163.com (D.Z.); 18248028134@163.com (L.W.); lq211105@163.com (Q.L.); aori6009@163.com (A.C.); 2 College of Veterinary Medicine, Inner Mongolia Agricultural University, Hohhot 010018, China; chunjiewang200@sohu.com

**Keywords:** feed-to-gain, nutrient metabolism, cattle

## Abstract

To systematically investigate the metabolic and microbial differences in feed efficiency between Hereford × Simmental (H × S) crossbred and purebred Simmental cattle (S × S), 20 steers (initial weight = 268.80 ± 30.88 kg; initial age = 273.00 ± 14.09 days) were used, which were housed in mixed pens and fed a low-energy diet (0.75 Mcal Neg/kg DM). This study revealed that the level of Hypoglycin B was related to a reduced T3, which could affect growth performance. Metabolomics analysis identified Hypoglycin B, lysoPC, and DHAP as key discriminative metabolites. Pathway enrichment analysis revealed that these metabolites were primarily mapped to glycine, serine, and threonine metabolism; alanine, aspartate, and glutamate metabolism; and pantothenate and CoA biosynthesis. In our study, the relative abundances of Succinivibrio and Clostridium_sensu_stricto_6 were significantly greater in the H × S group than in the S × S group; it may have stronger energy extraction abilities and matches their lower F/G. Romboutsia was positively correlated with Hypoglycin B and negatively correlated with LysoPC (20:2 (11Z,14Z)/0:0), which might be related to changes in blood metabolites. Nevertheless, these explanations remain correlational and require functional validation through targeted interventions.

## Simple Summary

Improving feed efficiency is a key goal in sustainable beef production. By examining hormonal signals, blood metabolites, and gut microbes, this study investigated the differences in feed efficiency between Hereford × Simmental crossbred cattle and purebred Simmental cattle by analyzing hormone signals, blood metabolites, and gut microbes. We found that the level of Hypoglycin B was related to a reduced T3, which could affect growth performance. Metabolomics analysis identified Hypoglycin B, lysoPC, and DHAP as key discriminative metabolites. Pathway enrichment analysis revealed that these metabolites were primarily mapped to glycine, serine, and threonine metabolism; alanine, aspartate, and glutamate metabolism; and pantothenate and CoA biosynthesis. In our study, the relative abundances of Succinivibrio and Clostridium_sensu_stricto_6 were significantly greater in the H × S group than in the S × S group; it may have stronger energy extraction abilities and matches their lower F/G. Romboutsia was positively correlated with Hypoglycin B and negatively correlated with LysoPC (20:2 (11Z,14Z)/0:0), which might be related to changes in blood metabolites.

## 1. Introduction

Since the 1960s, China has introduced high-quality foreign beef cattle breeds, which are currently raised all over the country. By crossbreeding these breeds with our local yellow cattle, beef production has clearly improved [1]. Among the beef cattle breeds in China, those most commonly crossbred with our yellow cattle are Simmental, Charolais, Limousin, and Angus, with Simmental crossbreeds being the main type [2]. However, overall, the supply of high-quality breeding stock still does not meet the upgraded needs of industry. Therefore, the lack of high-quality specialized beef breeds is among the main bottlenecks limiting the improvement of China’s beef industry. This directly affects the quality structure of our beef products, since much of the beef comes from dual-purpose cattle, local yellow cattle, and culled dairy cows, resulting in insufficient production and uneven meat quality, which makes it difficult to meet consumers’ demand for high-quality, healthy beef [3]. Against this backdrop, optimizing beef cattle performance through crossbreeding has become a necessary choice for the efficient and healthy development of China’s beef industry.

In beef cattle production, feed costs account for approximately 60–75% of total operating expenses, making feed efficiency a critical determinant of both farm profitability and production system sustainability [4]. F/G is a core economic indicator for measuring beef cattle production performance. It directly reflects the efficiency of feed conversion and is closely related to farming costs and economic benefits. It not only affects the growth rate and slaughter performance of animals but also indirectly regulates intramuscular fat deposition and meat quality by changing how the body distributes energy [5]. The hindgut microbiota plays a key role in further breaking down feed residue and producing volatile fatty acids (VFAs), and its compositional differences are closely related to feed efficiency [6]. Research has shown that in high-feed-efficiency groups (low F/G), hindgut microbes present unique metabolic traits, such as increased expression of genes involved in carbohydrate metabolism and VFA production [7]. From a metabolic perspective, improvements in F/G are often accompanied by changes in the serum metabolite profile, including the reprogramming of metabolites related to amino acids, fatty acids, and energy metabolism [8]. Integrative analysis of the microbiome and metabolome further revealed that certain bacterial groups (such as *Lactobacillus* and *Blautia*) can influence the body’s energy metabolism and fat deposition by regulating the production of key metabolites such as propionate and butyrate [9]. Previous work by Omondi et al. revealed that feed efficiency in ruminants is intimately linked to differences in rumen microbial community composition [10,11]. However, research on how gut microbes affect host physiological and metabolic functions is still limited, although this topic is receiving increasing attention [12].

Therefore, integrating metabolomic and microbiomic data facilitates the construction of a multi-omics association map significantly correlated with feed efficiency variations from a systems biology perspective, thereby providing candidate biomarkers and potential intervention targets for optimizing beef cattle production performance.

## 2. Materials and Methods

This study was conducted in Chifeng, Inner Mongolia, China, and consent was obtained from the legal owners for the use of animals and private land. The sampling was approved by Inner Mongolia Agricultural University, Hohhot, China (protocol number 2020079; 21 November 2020), and the researchers confirmed that they followed the relevant requirements of the ARRIVE guidelines for in vivo animal research.

### 2.1. Animal Management and Sample Collection

In total, 20 steers (initial weight = 268.80 ± 30.88 kg; initial age = 273.00 ± 14.09 days) were used in this study, comprising 10 purebred Simmental (S × S) and 10 Simmental × Hereford crossbreds (H × S). All the experimental cattle were raised in mixed pens at the Shengquan Ecological Animal Husbandry Co., Ltd. farm in Chifeng, Inner Mongolia, and the feeding plan followed the same strategy (see Table 1). Before the formal experiment, there was a 15-day prefeeding period, followed by a 90-day trial period. They were fed a total mixed ration twice a day (08:00 and 18:00) and had free access to food and water. At the conclusion of the experiment on Day 90, blood and fecal samples were collected from the experimental cattle after they had fasted. The sampler wore disposable long-arm gloves and manually collected fresh fecal samples from the rectum of each animal. The serum, plasma, and fecal samples were aliquoted, immediately placed in liquid nitrogen for transport to the laboratory, and subsequently stored at −80 °C until further analysis [13]. The present design provides a terminal snapshot of endocrine, metabolomic, and microbial states rather than longitudinal characterization of adaptation dynamics.

### 2.2. Microbial Community Diversity Analysis of Rectal Contents

For microbiome analysis, fresh rectal contents were collected on Day 90 (n = 10), and microbial DNA was extracted according to the instructions of the E.Z.N.A. Soil DNA Kit (Omega Bio-Tek, Norcross, GA, USA). The quality and concentration of the DNA were determined using 1.0% agarose gel electrophoresis and a NanoDrop 2000 spectrophotometer (Thermo Scientific, Waltham, MA, USA), and the samples were stored at −80 °C until further use [14]. Sequencing was carried out by Majorbio Bio-Pharm Technology Co., Ltd., Shanghai, China. The V3-V4 hypervariable region of the bacterial 16S rRNA gene was amplified using an ABI GeneAmp^®^ 9700 PCR thermocycler (Applied Biosystems, Foster City, CA, USA) and paired-end sequenced on the Illumina NextSeq 2000 platform (Illumina, San Diego, CA, USA). The raw sequence data have been deposited in the SRA database and are publicly available under accession number PRJNA1465087 (https://www.ncbi.nlm.nih.gov/sra/PRJNA1465087) (accessed on 13 May 2026) as of the publication of this paper. The specific procedures followed the method described by He [15]. For the classification of ASVs and their descriptions, please refer to reference [16].

### 2.3. Plasma Metabolomic Profiling by LC–MS/MS

After fasting, blood was collected from each animal in anticoagulated blood collection tubes and allowed to stand for 30 min; the samples were subsequently centrifuged (80–2, Jiangsu Kangjie Medical Devices Co., Ltd., Taizhou, China) at 1076.7 rcf for 10 min to separate the plasma, which was then divided into centrifuge tubes and stored at −80 °C for LC–MS/MS analysis. The metabolites from the samples were extracted following the methods of Chen et al. [13]. The resulting supernatant was transferred to sample vials for instrumental analysis. To monitor the stability of the analytical system, equal aliquots from all the samples were pooled to create a quality control (QC) sample. The QC material was processed and injected in the same manner as the study samples at intervals of every 5–15 injections. Chromatographic separation was carried out on a Thermo UHPLC-Q Exactive HF-X system fitted with an ACQUITY HSS T_3_ column (100 × 2.1 mm, 1.8 μm; Waters, Milford, MA, USA) at Majorbio Bio-Pharm Technology Co., Ltd. (Shanghai, China), following the procedures described by Yang et al. [16] and Chen et al. [13].

### 2.4. Determination of Blood Indices

All serum biochemical indices were measured in triplicate using bovine-specific ELISA kits (Ruixin Biotechnology, Quanzhou, China) according to the manufacturer’s protocols [17]. For each sample, if the coefficient of variation (CV) among the three replicates exceeded 10%, the replicate with the greatest deviation from the mean was excluded, and the remaining two replicates was used for statistical analysis. We measured the concentrations of FFAs, glucagon (GC), growth hormone (GH), gonadotropin-releasing hormone (GnRH), insulin (INS), leptin (LEP), T_3_, thyroxine (T_4_), TG, TRH, TSH, low-density lipoprotein (LDL), high-density lipoprotein (HDL) and very low-density lipoprotein (VLDL).

### 2.5. Data Statistics and Analyses

Statistical analyses were performed using SPSS 24.0. Serum biochemical parameters, F/G, initial body weight, final body weight, and ADG were analyzed using independent-samples ANOVA, and statistical significance was determined by independent-samples ANOVA. The results of this study are presented as the means ± standard deviations (SD), and *p* < 0.05 was considered statistically significant. F/G = DMI/ADG. On the 45th day of the experiment, the DMI value was measured according to Chen’s method over three consecutive days [18]. To avoid the confounding effects of heat stress on feed intake, DMI measurements were conducted prior to the onset of heat stress. Specifically, individual feed intake was recorded continuously for 3 days during the mid-experimental period (Day 45), when ambient temperature remained within the thermoneutral zone (daily mean temperature < 25 °C and THI < 72). The average DMI over this 3-day window was used as the representative feed intake value for 5 cattle throughout the entire experimental period, as preliminary observations indicated that within-animal variation in DMI during the pre-heat-stress phase was minimal (coefficient of variation < 5%). Owing to space limitations, we measured the DMI of 5 cattle, which is somewhat limiting.

## 3. Results

As shown in Table 2, there were no significant differences in body weight at 0 d, body weight at 90 d, or ADG among the test cattle. The F/G of the cattle in the H × S group was significantly lower than the S × S group (*p* < 0.05).

As shown in Table 3, compared with those in the S × S group, the T3 and the FFA contents in the S × S group were significantly lower (*p* < 0.05). There were no significant differences in the other indicators between the two groups.

**Figure 1:**
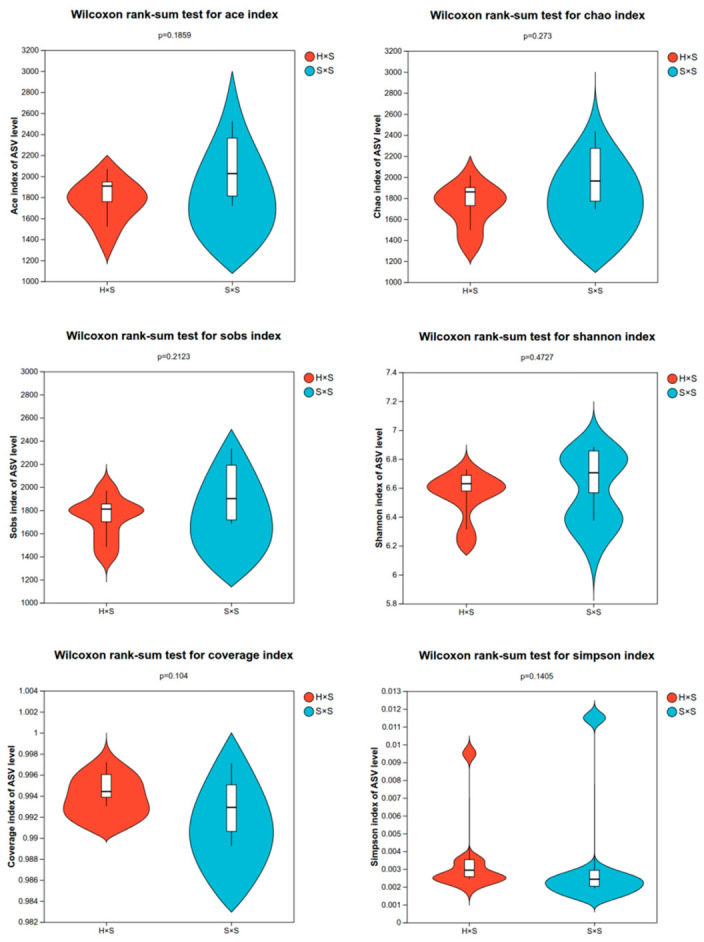
Differences in the α diversity between the two groups.

**Figure 2:**
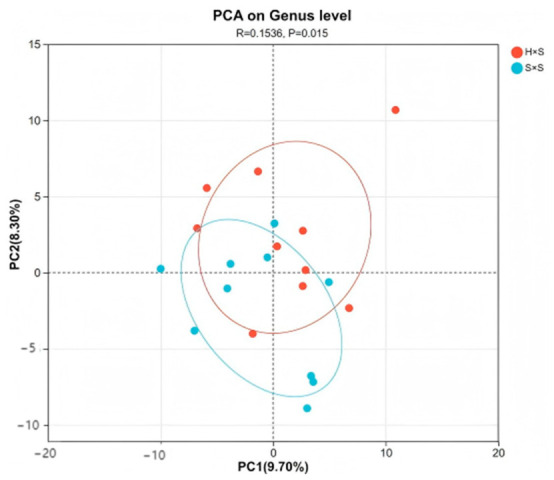
Differences in the β diversity between the two groups.

As is evident from Figure 1, none of the α diversity indices reached significance (*p* > 0.05), suggesting that the diversity of the gut microbiota did not substantially differ between the two groups of cattle. In contrast, the β diversity analysis (Figure 2) revealed that within the 95% confidence interval, the flora of the two groups of experimental cattle could be distinctly differentiated (*p* < 0.05). Notably, these findings suggest significant changes in the structure of the gut microbiota.

**Figure 3:**
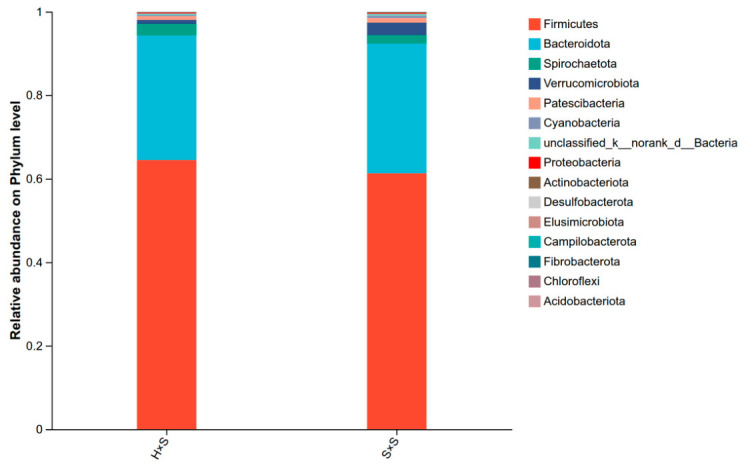
Bar chart of the relative abundance of species at the phylum level in the two groups.

**Figure 4:**
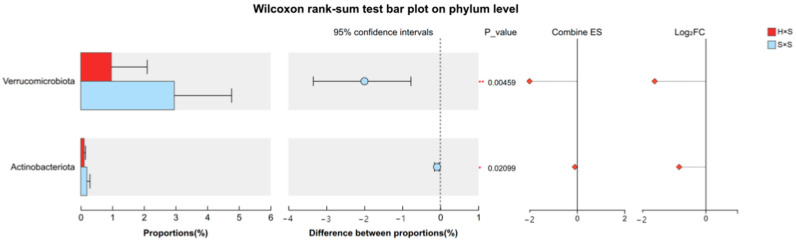
Phylum-level differences between the two groups. * indicates a significant difference between the two groups and ** indicates a highly significant difference between the two groups.

As shown in Figure 3, a phylum-level microbial community analysis of the gut microbiota was conducted. The sample means were calculated for each group. Among all the valid sequences from the two groups, five dominant phyla (Firmicutes, Bacteroidetes, Spirochaetes, Verrucomicrobiota, and Patescibacteria) were detected, whereas the relative abundance of each of the remaining phyla was less than 1%. In the hindgut of the experimental cattle (in Figure 4), the relative abundance of Verrucomicrobiota (*p* < 0.05) and Actinobacteriota (*p* < 0.05) in the S × S group was significantly greater than that in the H × S group.

**Figure 5:**
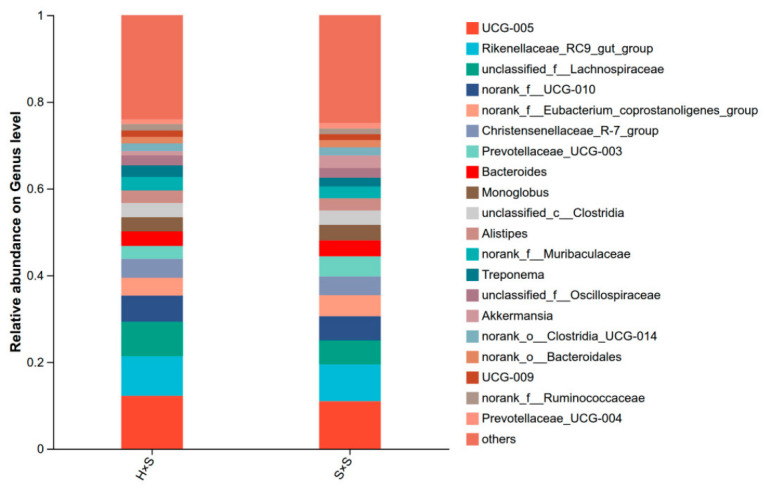
Bar chart of the relative abundance of species at the genus level in the two groups.

**Figure 6:**
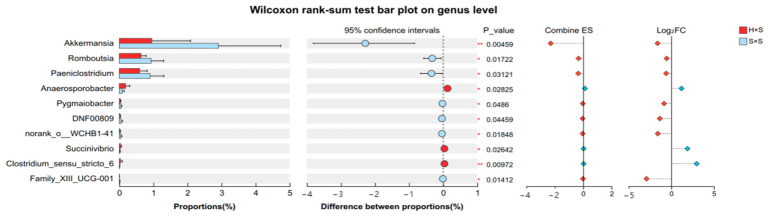
Genus-level differences between the two groups. * indicates a significant difference between the two groups and ** indicates a highly significant difference between the two groups.

As shown in Figure 5, 20 bacterial genera whose relative abundance was >1% were identified in rectal content samples from experimental cattle. The dominant genera included *Ruminococcaceae UCG-005*, the *Rikenellaceae RC9* gut group, the *Christensenellaceae R-7* group, *Prevotellaceae UCG-003*, and *Bacteroides*. As shown in Figure 6, the relative abundances of *Anaerosporobacter*, *Succinivibrio*, and *Clostridium-sensu-stricto-6* in the H × S group were significantly greater than those in the S × S group (*p* < 0.05); in addition, the relative abundances of *Akkermansia*, *Romboutsia*, *Paeniciostridium*, *DNF00809*, and *Family-XIII-UCG-001* in the S × S group were significantly greater than those in the H × S group (*p* < 0.05).

**Figure 7:**
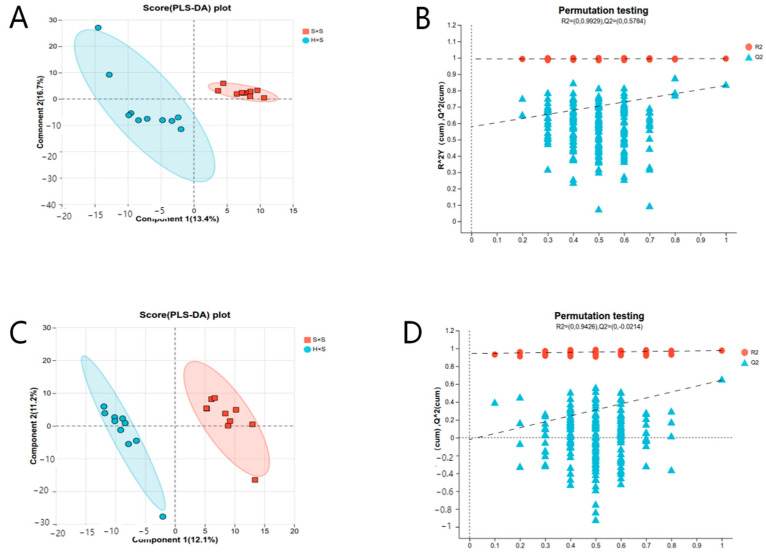
Corresponding validation plots of orthogonal projections to PLS-DA models (**A**,**C**) and permutation tests of the OPLS-DA models (**B**,**D**).

Score plots of the PLS-DA results are shown in Figure 7A,C, and the OPLS-DA results are shown in Figure 7B,D. The different metabolites identified in the two groups were verified. The R2Y values of POS and NEG were 0.9929 and 0.9426, respectively. Panels (A,B) and (C,D) represent the POS group and the NEG group, respectively. In Figure 7A,C, red and blue represent the comparison results of the S × S and H × S groups, respectively, with those of the control cattle (n = 10).

**Figure 8:**
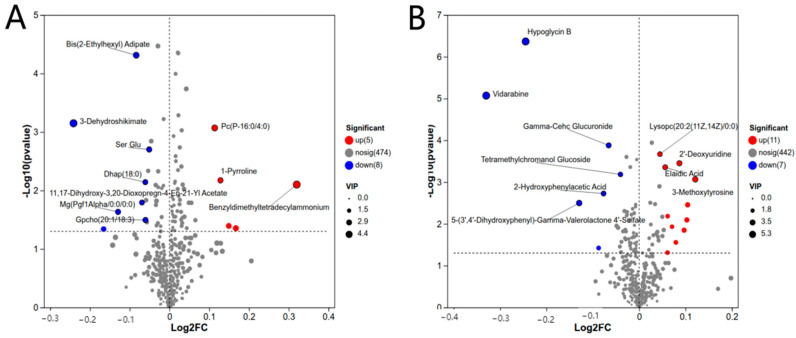
Volcano plot showing the differentially abundant metabolites of the H × S group vs the S × S group in POS (**A**) and NEG (**B**) ion modes. Red and blue represent upregulation and downregulation, respectively, whereas grey indicates no significant difference. *p*-Values of each metabolite from the differential analysis are shown on the *y*-axis.

The various metabolites between the two groups are clearly shown in Figure 8, and the top 10 metabolites ranked by *p* value are labelled. Detailed information on these metabolites is provided in Table 4. Additionally, the concentrations of Hypoglycin B, DHAP (18:0), and GpCho decreased in the H × S group. The concentrations of LysoPC(20:2(11Z,14Z)/0:0), elaidic acid, and Pc(P-16:0/4:0) were *greater* in the H × S group.

**Figure 9:**
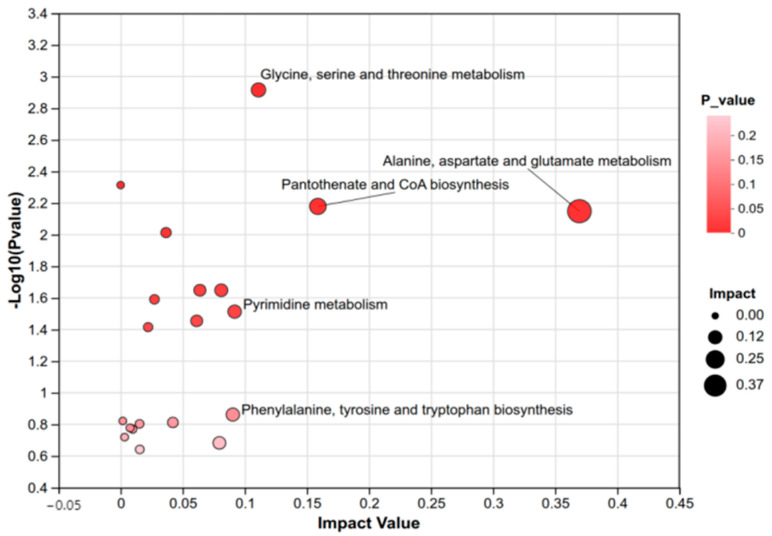
Enrichment analysis of differentially abundant metabolites between the two groups of cattle. The *x*-axis shows the pathway impact values from the topology analysis; the *y*-axis shows the *p*-values of the metabolic pathways from the enrichment analysis.

As shown in Figure 9, the metabolites identified in plasma are involved in alanine, aspartate, and glutamate metabolism; glycine, serine, and threonine metabolism; and pantothenate and CoA biosynthesis, which were significantly enriched in the H × S group and S × S group (Figure 9).

**Figure 10:**
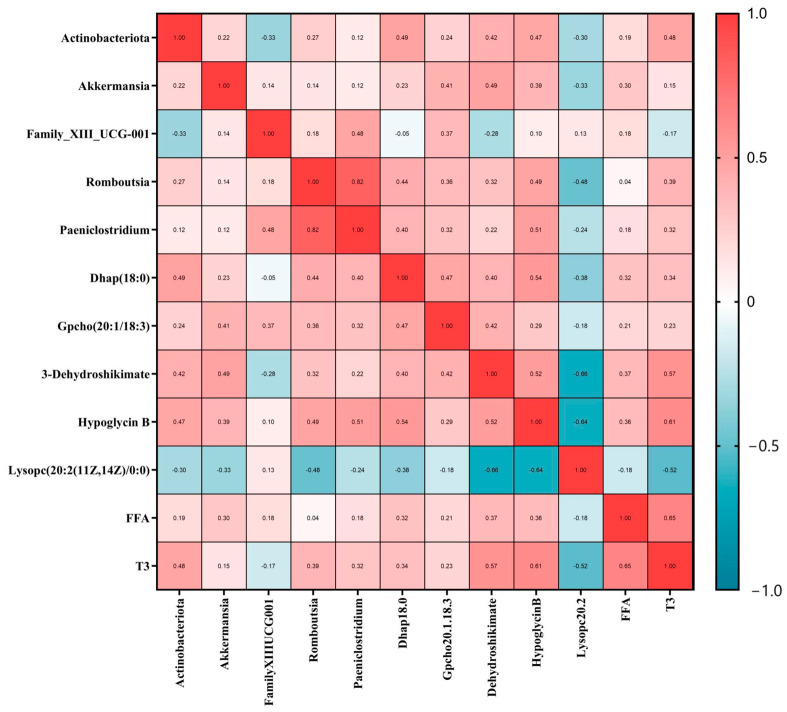
Correlation analysis between several differential hormones, blood metabolites and gut microbes.

In the bovine gut, Verrucomicrobiota was entirely assigned to *Akkermansia*; thus, only the genus-level data were used for correlation analysis. Correlation analysis revealed that cattle serum T3 was significantly positively correlated with FFAs (r = 0.65; *p* < 0.01), that Hypoglycin B was significantly positively correlated with T3 levels (r = 0.61; *p* < 0.01), that LysoPC was negatively correlated with T3 and Hypoglycin B (r = 0.52; *p* < 0.05; r = 0.64; *p* < 0.01), and that LysoPC was significantly negatively correlated with T3 and Hypoglycin B (r = 0.52; *p* < 0.05; r = 0.64; *p* < 0.01). *Romboutsia* was positively correlated with Hypoglycin B (r = 0.49, *p* < 0.05) (Figure 10).

## 4. Discussion

The FFA levels in the H × S group of cattle decreased significantly, indicating reduced fat breakdown. Moreover, T_3_ levels decreased significantly, whereas T_4_, TSH, and TRH levels did not significantly change. T_3_ plays a central role in regulating metabolic efficiency and energy expenditure [19]. Lower circulating T_3_, which correlates with a suppressed basal metabolic rate, reduces maintenance energy requirements. This sparing effect potentially increases the net energy available for productive functions, although this partitioning depends on the physiological state and nutrient supply [20]. In our study, correlation analysis revealed that bull serum T_3_ levels were significantly positively correlated with FFA levels (r = 0.65, *p* < 0.01). In healthy animals, changes in T_3_ and FFA tend to occur in the same direction [21]. The lower T_3_ aligns with reduced FFA, which may help shift energy towards muscle deposition and optimize growth performance. This finding is consistent with our trial results, where the F/G ratio in the H × S group was significantly greater than that in the S × S group.

The results of the metabolomic analysis in the present study found that serum Hypoglycin B levels were significantly lower in the H × S group than in the S × S group. Hypoglycin is a naturally occurring amino acid derivative found in the ackee fruit (*Blighia sapida*) and is known to inhibit fatty acid β-oxidation by interfering with the activity of acyl-CoA dehydrogenases, thereby blocking mitochondrial fatty acid oxidation and leading to hypoglycaemia and lipid accumulation [22]. The results of the correlation analysis in this study revealed that Hypoglycin B was significantly positively correlated with T_3_ levels (r = 0.61, *p* < 0.01). Therefore, the Hypoglycin B content in the H × S group may be related to a reduced T_3_, enabling efficient energy redistribution, which in turn is associated with growth performance. In addition, our data also revealed that in the H × S group, the intermediate metabolites involved in liver lipid synthesis—DHAP (18:0) and glycerophosphocholine (GpCho)—were both downregulated. GpCho is an important intermediate in membrane phospholipid metabolism and serves as an indicator of the regulation of lipid metabolic homeostasis [23]. As an intermediate in glycolysis, dihydroxyacetone phosphate (DHAP) is the precursor of the glycerol backbone in triglyceride biosynthesis; its decreased level likely reflects that fat formation in the H × S group is suppressed. Lysophosphatidylcholine (LysoPC) is involved in lipid signalling and the regulation of insulin sensitivity [24]. Correlation analysis revealed that LysoPC was negatively correlated with T_3_ and Hypoglycin B (r = 0.52, *p* < 0.05; r = 0.64, *p* < 0.01). Notably, in the H × S group, the expression of LysoPC (20:2 (11Z, 14Z)/0:0) increased significantly. In bull serum, LysoPC was significantly negatively correlated with T_3_ and Hypoglycin B (r = 0.52, *p* < 0.05; r = 0.64, *p* < 0.01). LysoPC, a key intermediate in glycerophospholipid metabolism, can activate the mitogen-activated protein kinase (MAPK) pathway and promote cell proliferation [25]. T_3_ is an important hormone for regulating protein deposition, and changes in nutritional levels can affect T_3_ production by regulating peripheral 5′-deiodinase activity, thereby influencing muscle growth efficiency [26,27]. So, LysoPC might be linked to lower fat mobilization. Additionally, KEGG enrichment analysis indicated that these differentially abundant metabolites are involved in glycine, serine, and threonine metabolism; alanine, aspartate, and glutamate metabolism; and pantothenate and CoA biosynthesis. The glycine, serine, and threonine metabolism pathway serves as a central hub for amino acid metabolism, one-carbon metabolism, and redox homeostasis [28]. Locasale et al. identified glycine and threonine as key metabolites that can be used to predict feed efficiency phenotypes. Pantothenate is a precursor of coenzyme A (CoA), an essential cofactor for fatty acid oxidation and the tricarboxylic acid (TCA) cycle [29]. In recent years, researchers have used combined metabolomics and microbiome analyses, linking changes in metabolites with microbial species, to offer new insights into how gut microbes regulate the body’s metabolism [30].

The gut microbiota coexists with the host and plays important roles in regulating digestion, gut development, nutrient absorption, and metabolism [31]. The gut microbiota is regarded as the ‘second genome’ of ruminants, significantly impacting various physiological functions and strongly influencing host metabolism and health [32,33,34]. In our study, the relative abundances of *Succinivibrio* and *Clostridium_sensu_stricto_6* were significantly greater in the H × S group than in the S × S group. As important succinate-producing bacteria in the gut of ruminants, *Succinivibrio* can produce succinate and activate gluconeogenesis in the ruminant gut, where they participate in the production of propionate [35]. The increased proportions of the Succinivibrio and Clostridium_sensu_stricto_6 populations suggest that the H × S group of cattle may have stronger carbohydrate fermentation and energy extraction abilities, which matches their lower feed conversion ratio (F/G). Similarly, the high abundance of *Clostridium_sensu_stricto* might be related to its enhanced ability to produce butyrate. [36]. By serving as the favoured oxidative fuel of colonocytes, butyrate reinforces gut barrier function and lessens the energy drain imposed by inflammatory processes [36]. The gut microbiota has been shown to play a key role in regulating feed efficiency through multiple mechanisms, including the modulation of intestinal architecture, immune function, and host energy homeostasis [37]. In summary, the relative abundance of *Akkermansia* increased within the S × S group. *Akkermansia* is specialized for mucin degradation in the intestinal mucus layer; however, it concurrently stimulates the host to replenish mucin, an interplay that may support mucosal barrier preservation and metabolic well-being [38]. In this study, the relative abundance of *Romboutsia* in the H × S group was significantly lower than that in the S × S group. *Romboutsia* has been shown to improve lipid profiles and body metabolism [39]. Correlation analysis revealed that *Romboutsia* was positively correlated with Hypoglycin B (r = 0.49, *p* < 0.05) and negatively correlated with LysoPC (20: 2 (11Z, 14Z)/0: 0) (r = 0.48, *p* < 0.05), which might be related to changes in blood metabolites. A notable limitation of this study is the small sample size (n = 5) for DMI measurements, owing to farming environment constraints. This limits the statistical power to detect subtle associations and may affect the generalizability of our findings. Therefore, independent validation in larger cohorts with precisely recorded individual feed intake and hindgut fermentation parameters is essential to confirm the utility of these candidate markers.

## 5. Conclusions

Our study reveals variations in hindgut microbiota composition and metabolism in cattle. This study revealed that the level of Hypoglycin B was related to a reduced T_3_, which could affect growth performance. Metabolomics analysis identified Hypoglycin B, lysoPC, and DHAP as key discriminative metabolites. Pathway enrichment analysis revealed that these metabolites were primarily mapped to glycine, serine, and threonine metabolism; alanine, aspartate, and glutamate metabolism; and pantothenate and CoA biosynthesis. In our study, the relative abundances of Succinivibrio and Clostridium_sensu_stricto_6 were significantly greater in the H × S group than in the S × S group; it may have stronger energy extraction abilities and matches their lower F/G. *Romboutsia* was positively correlated with Hypoglycin B and negatively correlated with LysoPC (20:2 (11Z,14Z)/0:0), which might be related to changes in blood metabolites. Nevertheless, these explanations remain correlational and require functional validation through targeted interventions.

## Figures and Tables

**Table 1:** Composition and nutrient levels (dry matter basis) of the experimental diets.

Components	Proportion (%)
DM	46.9
ADF	30.3
NDF	43.8
CP	14.2
Lignin	4.9
Fat	2.4
Ash	9.6
Ca	0.77
P	0.43
Mg	0.40
K	1.42
NEg, Mcal/kg	0.75
ME, Mcal/kg	2.37
Ingredients(%)	
Corn stover	16.67
Oat hay	20.83
Corn silage	25.00
Corn	17.80
Wheat bran	8.40
Soybean meal	9.50
CaHPO_4_	0.30
NaCl	0.50
Premix ^1^	1.00
Total	100.00

^1^ The premix contained (per kg premix) 370,000 IU vitamin A, 100,000 IU vitamin, D3, 5000 IU vitamin E, 3000 mg of Zn, 2500 mg Fe, 1680 mg of Mn, 780 mg of Cu, 150 g of Ca, 30 mg of I, 30 mg of Se, 26 g of P, and 20 mg of Co.

**Table 2:** Production performance of the cattle in the two groups.

Item	H × S	S × S	*p*-Value
Body Weight at 0 d (kg, n = 10)	271.95 ± 30.65	265.65 ± 32.42	0.661
Body Weight at 90 d (kg, n = 10)	323.95 ± 40.44	313.15 ± 38.23	0.547
ADG (kg, n = 10)	0.58 ± 0.25	0.53 ± 0.09	0.553
DMI (kg/d, n = 5)	9.02 ± 0.33	9.38 ± 0.17	0.062
F/G (n = 5)	15.77 ± 1.84	18.82 ± 1.42	0.046

**Table 3:** Serum metabolites of the cattle in the two groups.

Item	H × S	S × S	*p*-Value
FFA (µmol/L)	245.4003 ± 15.20543	261.2284 ± 23.00188	0.014
GC (ng/L)	57.7769 ± 8.99769	57.0716 ± 5.95371	0.772
GH (µg/L)	30.1999 ± 7.34183	31.4343 ± 6.40631	0.574
HDL (µmol/L)	618.3208 ± 247.35008	580.4158 ± 255.48313	0.636
GnRH (ng/L)	30.1429 ± 3.98111	31.5688 ± 3.56277	0.240
INS (mIU/L)	21.5711 ± 2.04402	22.128 ± 2.69509	0.466
LDL (µmol/L)	861.2502 ± 72.96546	875.9297 ± 70.97975	0.523
LEP (ng/L)	2590.0001 ± 225.52375	2588.2112 ± 216.55851	0.980
T_3_ (pmol/L)	44.6276 ± 5.12739	54.7997 ± 9.27522	0.000
T_4_ (pmol/L)	337.0282 ± 79.7434	376.1366 ± 91.72617	0.158
TG (µmol/L)	413.5283 ± 22.09324	418.3621 ± 21.00275	0.483
TRH (pg/mL)	39.3845 ± 8.54475	38.6603 ± 3.81346	0.731
TSH (µIU/L)	436.8627 ± 62.12588	413.1569 ± 32.35164	0.138
VLDL (µg/mL)	29.318 ± 6.38157	29.0771 ± 3.25196	0.881

**Table 4:** Plasma metabolites of the cattle in the two groups.

Metabolite	VIP	FC (H × S/S × S)	*p*-Value	Positive/Negative	M/Z
Vidarabine	5.3037	0.7957	<0.00001	neg	312.0963
Hypoglycin B	5.2204	0.8439	<0.00001	neg	269.1153
3-Dehydroshikimate	4.3962	0.8459	0.0007106	pos	204.063
Benzyldimethyltetradecylammonium	4.3042	1.2485	0.007932	pos	332.3313
5-(3′,4′-Dihydroxyphenyl)-Gamma-Valerolactone 4′-Sulfate	3.1639	0.9143	0.003176	neg	269.0135
Pi(Pgj2/20:1(11Z))	3.0578	1.1228	0.04422	pos	975.5891
Pc(P-16:0/4:0)	2.9458	1.0821	0.0008536	pos	572.3716
3-Methoxytyrosine	2.9442	1.0874	0.0008606	neg	210.0771
Bis(2-Ethylhexyl) Adipate	2.8983	0.9437	0.0000488	pos	371.3155
Uridine	2.7817	1.0752	0.003498	neg	279.0397
N-(3-Aminopropyl)-N-Methylcarbamic Acid Tert-Butyl Ester	2.7691	1.109	0.0404	pos	189.1596
(10E,12E,14E)-16-Hydroxy-9-oxooctadeca-10,12,14-trienoylcarnitine	2.7052	1.074	0.008055	neg	450.2883
2′-Deoxyuridine	2.7023	1.062	0.0003545	neg	227.0676
Glycolithocholic Acid	2.6633	1.0695	0.01417	neg	432.3136
Gamma-Cehc Glucuronide	2.6032	0.9554	0.0001321	neg	421.1524
Tributyl Phosphate	2.5351	0.8915	0.04576	pos	267.1719
Elaidic Acid	2.3871	1.0397	0.0004399	neg	327.255
2-Hydroxyphenylacetic Acid	2.3683	0.9482	0.001878	neg	151.0397
Ser Glu	2.3565	0.9653	0.001994	pos	235.0923
1-Pyrroline	2.3223	1.093	0.006717	pos	70.0655
Mg(Pgf1Alpha/0:0/0:0)	2.3059	0.9142	0.02325	pos	448.3266
Gpcho(20:1/18:3)	2.3017	0.959	0.03207	pos	832.5836
Demethoxyfumitremorgin C	2.2575	0.9413	0.03776	neg	370.1521
Lovastatin Acid	2.2343	1.0564	0.02775	neg	421.2612
Lysopc(20:2(11Z,14Z)/0:0)	2.1927	1.0315	0.0002144	neg	592.3648
N-Palmitoyl Tyrosine	2.1532	1.0433	0.0487	neg	464.3035
Dhap(18:0)	2.1274	0.9587	0.007211	pos	500.275
4-Ethylphenol	2.1266	1.0435	0.006544	neg	121.0654
Tetramethylchromanol Glucoside	2.0642	0.9725	0.0006524	neg	427.1992
11,17-Dihydroxy-3,20-Dioxopregn-4-en-21-yl Acetate	2.0293	0.9532	0.01609	pos	446.2541
Kitaguni	2.0259	1.0504	0.01169	neg	241.0446

## Data Availability

The raw sequence data have been deposited in the SRA repository and are publicly available under the accession number PRJNA1465087 as of the date of publication (https://www.ncbi.nlm.nih.gov/sra/PRJNA1465087) (accessed on 13 May 2026).
